# Adherence to the Mediterranean Diet Is Inversely Associated with the Prevalence of Metabolic Syndrome in Older People from the North of Spain

**DOI:** 10.3390/nu14214536

**Published:** 2022-10-28

**Authors:** Gloria Cubas-Basterrechea, Iñaki Elío, Guzmán Alonso, Luis Otero, Luis Gutiérrez-Bardeci, Jesús Puente, Pedro Muñoz-Cacho

**Affiliations:** 1 Dietetic Section, Hospital Universitario “Marqués de Valdecilla”, 39008 Santander, Spain; 2 Research Group on Foods, Nutritional Biochemistry and Health, Universidad Europea del Atlántico, 39011 Santander, Spain; 3 Department of Health, Nutrition and Sport, Iberoamerican International University, Campeche 24560, Mexico; 4 Primary Care Center Vargas, 39010 Santander, Spain; 5 Primary Care Center Puertochico, 39004 Santander, Spain; 6 Compass Group, Department VITArest, 28054 Madrid, Spain; 7 Teaching Department of Primary Care Management, Cantabrian Health Service, IDIVAL, 39011 Santander, Spain

**Keywords:** diet, Mediterranean, metabolic syndrome, aged, Spain

## Abstract

Background: The aim of this study was to relate adherence to the Mediterranean diet (MedDiet) to the prevalence of metabolic syndrome (MetS) in an elderly population from the north of Spain. Methods: We carried out an observational, descriptive, cross-sectional, and correlational study involving 556 non-institutionalised individuals aged 65 to 79 years. The MEDAS-14 questionnaire score was used to define the degree of adherence to the Mediterranean diet. The diagnosis of MetS was conducted using the International Diabetes Federation (IDF) criteria. Results: In 264 subjects with an average age of 71.9 (SD: ±4.2), 39% of whom were men, 36.4% had good adherence (score ≥ 9 in MEDAS-14), with no differences by gender or age. The prevalence of MetS was 40.2%, with 47.6% in men and 35.4% in women (*p* < 0.05). The prevalence of MetS was 2.4 times more frequent among individuals who consumed less than two servings (200 g) of vegetables daily compared with those who consumed two or more servings of vegetables daily (OR: 2.368, 95%CI: 1.141–4.916, *p* = 0.021). Low adherence to the MedDiet (MEDAS-14 score ≤ 8) was associated with an 82% higher prevalence of MetS (OR: 1.817, 95%CI: 1.072–3.081, *p* = 0.027). Conclusion: An inverse relationship was established between adherence to the MedDiet and the prevalence of MetS.

## 1. Introduction

The European Union (27 countries) is considered the area of the world with the second-highest percentage of elderly people (20.8%) [1] after Japan (28.4%) [2]. Italy (23.6%), Finland (22.7%), and Greece (22.6%) are the highest-ranking EU countries in terms of their aging population [1]. In Spain, 19.8% of the population is 65 years of age or older [1] and, specifically in Santander (Santander, Spain), located in the north of Spain, this percentage reached 25.7% in 2022 [3]. All of these data indicate that the elderly population group is of great importance and their dietary habits should be studied in depth, as they may have a decisive impact on healthy aging.

One way to analyse dietary habits is to determine adherence to the Mediterranean Diet (MedDiet), which is a healthy dietary pattern. In 2010, it was declared “Intangible Heritage of Humanity” by UNESCO [4]. Scientific evidence has supported its consumption by the elderly due to its benefits and effectiveness related to longevity, quality of life, and disease prevention [5]. Compliance with the MedDiet involves following a series of recommendations, such as the use of olive oil as the main source of fat for cooking; consuming foods of plant origin in abundance and frequency (fruits, vegetables, legumes, nuts); consumption of whole grains (bread, pasta, rice); daily consumption of dairy products; and consumption of fish, eggs, and white meats on a regular weekly basis, while red and processed meats should be consumed occasionally and wine in moderation, with water being considered the main drink [6]. In addition, the foods included in the MedDiet bring beneficial qualitative characteristics to this diet since they are rich in bioactive components such as antioxidants (vitamins C and E, among others), fibre, and phytosterols (from vegetables, fruits, legumes, nuts, whole grains, olive oil, and wine), and provide the correct balance (between 2:1 and 1:1) of polyunsaturated fatty acids (omega-6 vs. omega-3) through regular consumption of fish, seafood, and nuts, along with high consumption of monounsaturated fats (through daily use of olive oil) and low consumption of saturated fats (e.g., butter and red meat) [7]. Greater adherence to the MedDiet allows—taking into account its components as a whole and through multiple mechanisms—for improved glucose metabolism, reduction in blood pressure, a better lipid profile, and decreased serum markers of inflammation and oxidation [8,9]. As such, the MedDiet plays an important role in the prevention and treatment of pathologies related to chronic inflammation, such as metabolic syndrome (MetS), type 2 diabetes mellitus (DM2), and cardiovascular disease (CVD) [10,11].

MetS involves a set of risk factors associated with abdominal obesity and insulin resistance that are characterised by elevated blood pressure, hyperglycaemia, and lipid abnormalities, such as hypertriglyceridemia and low HDL-c. These metabolic and inflammatory alterations are a set of signs and symptoms that have a situation of hyperinsulinemia and insulin resistance in common, which imply a high risk of developing CVD and DM2 [12]. 

The aim of this study was to relate MedDiet adherence to the prevalence of MetS in an elderly population in northern Spain. 

## 2. Materials and Methods

### 2.1. Study Design

In this observational, descriptive, cross-sectional, and correlational study, we evaluate adherence to the Mediterranean Diet in order to analyse whether there existed an association with the prevalence of MetS in a population of elderly people.

### 2.2. Participants

The study population was non-institutionalised elderly persons aged 65 to 79 years, belonging to the quota of four doctors in three primary care centres (PCCs) in Santander (Cantabria) of the Cantabrian Health Service (CHS). According to data published by the Cantabrian Institute of Statistics (ICANE) on 31 January 2022, the population with these characteristics amounted to 30,295 individuals [3]. For the calculation of the sample number, 134 individuals with low adherence to the MedDiet (MEDAS-14 score ≤ 8) and 134 individuals with good adherence to the MedDiet (MEDAS-14 score ≥ 9) were required to detect a minimum relative risk of 2 if the rate of individuals with MetS in the group with good adherence to the MedDiet was 15%, assuming an alpha risk of 5% and a beta risk of 20%. A loss-to-follow-up rate of 0% was estimated. The Poisson approximation was used, and the Granmo v.7.12 program for finite populations [13] was used to calculate the representative sample. 

To obtain this sample, three-layer sampling was performed. First, the three PCCs in Santander with the largest number of patients over 65 years of age were chosen. Subsequently, a medical quota was chosen from each of the three centres on a purposive basis; that is, the chance to participate was offered to the coordinator of each of these PCCs and, from the centre that had the largest number of patients in this age group, a second medical quota proposed by the PCC coordinator was chosen. Finally, a random sample of individuals, stratified by gender and age (65–79 years), was chosen through systematic sampling.

**Figure 1:**
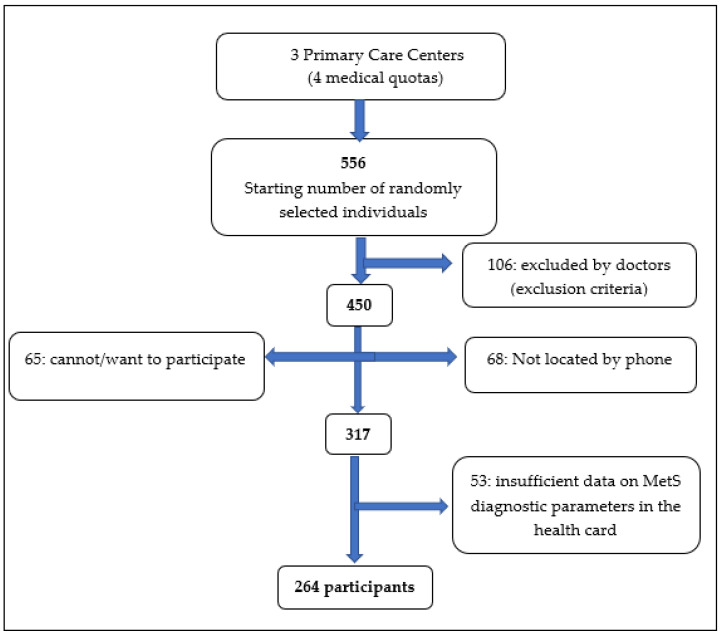
Flow chart of the participant selection process.

The fieldwork ran from 9 February to 16 September 2019 and began with 556 individuals. Of these, 239 individuals were ineligible since they were excluded by the doctors for meeting the following exclusion criteria: neurological, psychiatric, or psychological pathologies; physical problems with stability; mental and cognitive impairment (Pfeiffer test > 4 errors) [14]; and weight change (an increase/decrease of greater than 10%) in the last 12 months; or because they could not be located or were unable or unwilling to participate in the study. Of the resulting 317 individuals, there were 53 individuals who were not selected due to missing blood biochemical and/or medication analysis data from the last 12 months of MetS diagnostic parameters, according to the International Diabetes Federation (IDF) criteria [15]. Therefore, after random and systematic sampling, and after applying the selection and substitution criteria, a final sample of 264 participants (men, 39%; women, 61%) was obtained (see Figure 1).

### 2.3. Sociodemographic Variables

Sociodemographic characteristics were analysed, including gender, age, marital status, type of cohabitation, level of education, and the person who cooks. The following three age groups were established: 65–69 years, 70–74 years, and 75–79 years. Marital status included the following four levels: married/partnered, separated, widowed, and single. Types of cohabitation were divided into couple, with relatives, with a carer, alone, or in a shared flat. Educational levels were classified as university, secondary school, primary school, or incomplete primary school. The person who cooks was classified as the partaker, the couple, relatives, the carer, or catering (Table S1).

### 2.4. Body Mass Index Levels 

The BMI was calculated as the quotient between weight and height squared. According to the SEEDO 2000 classification [16], the following three levels of nutritional status were established according to their corresponding BMI thresholds: normal weight (18.5–24.9 kg/m^2^), overweight (25–29.9 kg/m^2^), and obese (30–49.9 kg/m^2^).

### 2.5. Diagnosis of Metabolic Syndrome

To make a diagnosis of MetS, the IDF criterion [15] was used, according to which abdominal obesity is an essential diagnostic parameter (waist circumference for European individuals: ≥94 cm in men and ≥80 cm in women). In addition, two or more of the following parameters must be present: arterial hypertension (AHT; ≥130/85 mmHg, being on treatment or diagnosed), fasting hyperglycaemia (≥100 mg/dL or previous diagnosis of DM2 or treatment), hypertriglyceridemia (≥150 mg/dL or on treatment), and low HDL-c (<40 mg/dL in men and <50 mg/dL in women or on treatment).

### 2.6. Instruments

#### 2.6.1. Adherence to the Mediterranean Diet

To determine the degree of adherence to the MedDiet, the MEDAS-14 questionnaire was used, which was validated for the elderly population (aged 55–80 years) at high cardiovascular risk [17]. It is composed of 12 questions regarding the frequency of consumption and two regarding intake habits characteristic of the Spanish MedDiet. From the sum of the values obtained for the 14 items, the degree of adherence to the MedDiet is determined, establishing four levels: high, 12–14; medium, 8–11; low, 5–7; and very low, <5. The authors of the questionnaire also considered another classification, with only two levels of adherence to the MedDiet according to the score obtained: ≥9, good adherence; and ≤8, low adherence [18]. This questionnaire, being short, facilitates its implementation in older individuals and also allows for the identification of parameters enabling dietary improvement towards a healthier diet.

#### 2.6.2. Body Mass Index Assessment

For the determination of the BMI, weight was measured with a SECA^®^711 scale model (SECA, Hamburg, Germany). Height was measured directly with a SECA^®^220 stadiometer (SECA, Hamburg, Germany) and a SECA^®^b213 portable model (SECA, Hamburg, Germany), or indirectly by measuring the knee–heel distance, measured with a stadiometer, using the formula of Chumlea et al. that relates age to knee height [19].

#### 2.6.3. Measurement of Diagnostic Parameters of Metabolic Syndrome

To measure waist circumference and, subsequently, determine the presence of abdominal obesity, a SECA^®^ model 203 (SECA, Hamburg, Germany) ergonomic tape with millimetric precision was used. For the diagnosis of AHT, blood pressure was measured with an OMRON M3^®^ comfort blood pressure monitor (Omron, Shimogyo-ku, Kyoto, Japan) with an automatic arm. It was also possible to use mentions on one’s health card of one or more drugs for the treatment of AHT. The diagnosis of fasting hyperglycaemia was made using fasting blood glucose values, and prescribed medications for DM2 were extracted from the health card. Hypertriglyceridemia was diagnosed through the results of triglyceride blood tests and medications included for this pathology on the health card. Low HDL-c could be diagnosed by means of the HDL-c blood data, which were obtained from the health card.

### 2.7. Statistical Analysis

SPSS software was used to analyse the data (released 2017, IBM SPSS Statistics for Windows, Version 25.0, IBM Corp., Armonk, NY, USA), as well as Epidat 4.2, July 2016 (Consellería de Sanidade, Xunta de Galicia, Santiago, Spain).

Qualitative variables were described by calculating frequencies and percentages. To establish the association between an independent variable (adherence to the MedDiet, adherence components in MEDAS-14, prevalence of MetS) and a dichotomous variable (gender) or more than one category (age group), Pearson’s chi-square statistical test was performed. If the qualitative variable had several categories that did not have an order (BMI, marital status, type cohabitation), in order to compare the results by gender or age groups, the chi-square test of goodness of fit was used in the Epidat 4.2 program. If the qualitative variable with several categories had an order (number of variables for diagnosis of MetS), a chi-square test of the trend allowed us to calculate whether there was any statistically significant difference by gender or age group in each category.

In the case of quantitative variables, the Kolmogorov–Smirnov (K–S) non-parametric test of normality was used to determine whether the distribution was normal. Variables with a normal distribution (age, waist circumference) were described using the mean and standard deviation (SD). To compare two categories (gender) and two independent groups, Student´s *t*-test was performed. To compare with more than two categories (age groups), the analysis of variance test (ANOVA) was performed.

Variables with a non-normal distribution (BMI, MEDAS-14 score) were described with the median and interquartile range (IQR). To compare two independent groups, the non-parametric Mann–Whitney test was performed. For comparison with a variable with more than two categories (age groups), a non-parametric Kruskal–Wallis test was conducted.

Finally, comparisons between variables were carried out to determine a possible association between the consumption of vegetables and the recommendations and adherence to the MedDiet with the prevalence of MetS. The odds ratio (OR) was used as a measure of association and was calculated using logistic regression. Furthermore, the OR was controlled in terms of possible confounding variables (gender and age groups).

## 3. Results

### 3.1. Adherence to the Mediterranean Diet

The median score obtained in the questionnaire was 8 (RIC: 3), which corresponded to a medium–low adherence to the MedDiet.

A total of 55.3% of the individuals had medium adherence to the MedDiet, followed by 37.9% with low adherence, with the lower percentages corresponding to very low (4.9%) and high (1.9%) adherence to the MedDiet. According to the classification of the results of the MEDAS-14 questionnaire using two levels, 36.4% had good adherence to the MedDiet. There were no statistically significant differences in these percentages by gender (Table S1) or age group.

**Figure 2:**
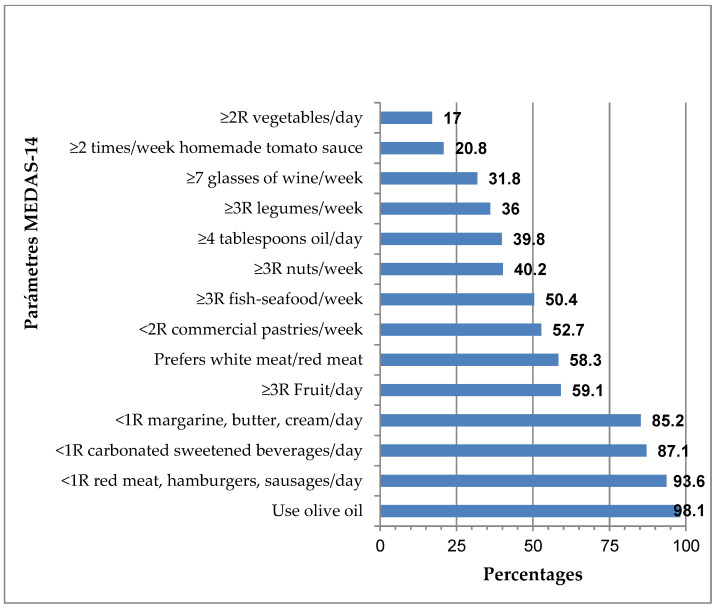
Adherence rates for the MEDAS-14 components.

The results obtained for the parameters analysed in the MEDAS-14 questionnaire are shown in Figure 2. The majority of individuals consumed olive oil for cooking (98.1%), although only 39.8% consumed four or more tablespoons of oil per day. At the other end of the scale, only 17.0% of the individuals consumed two or more servings of vegetables per day. It should be noted that more than 85% of the individuals consumed less than one serving per day of margarine, butter, and cream (85.1%); sugary carbonated beverages (87.1%); and red meat, hamburgers, or sausages (93.1%).

By gender, a higher percentage of women consumed two or more servings of vegetables daily (21.1%) in comparison to men (10.7%, *p* < 0.05), and were more likely to prefer to eat white meat instead of red meat (65.8%) in comparison to men (46.6%, *p* < 0.005). In contrast, men were more likely to consume seven or more glasses of wine per week (40.8%) compared with women (26.1%, *p* < 0.05; Table S1). By age group, there were no statistically significant differences in dietary pattern, except in the parameter of consumption of fewer than two servings of commercial pastries (not homemade) per week, which decreased with increasing age: between 65–69 years (63.6%), between 70–74 years (48.0%), and between 75–79 years (46.2%, *p* < 0.05).

### 3.2. Prevalence of Nutritional Status According to the Body Mass Index Levels

In the sample, 24.2% were normal weight, 50.0% overweight, and 25.8% obese (*p* = 0.001). By gender, men had a lower prevalence of normal weight (12.6%) than women (31.7%, *p* < 0.001). In contrast, men had a higher prevalence of overweight (60.2%) compared with women (43.5%, *p* < 0.01) and obesity (27.2%) compared with women (24.8%, no significant difference) (Table S2). No significant differences were found between the age groups.

### 3.3. Prevalence of Metabolic Syndrome

The prevalence of MetS was 40.2%. By gender, the prevalence in men (*n* = 103) was 47.6%, while that in women (*n* = 161) was 35.4%, with a statistically significant difference (*p* < 0.05; Table S2). The prevalence of MetS varied little between the different age groups, with no statistically significant differences.

The prevalence of abdominal obesity was 78.8%. Of the remaining MetS diagnostic variables, the prevalence of AHT and hyperglycaemia were the highest and significantly higher in men compared with women, as shown in Table S2. In addition, the majority of individuals had one (38.6%) or two (27.6%) MetS diagnostic variables other than abdominal obesity. By gender, males had higher percentages of two, three, and four MetS diagnostic variables; in contrast, women had a higher percentage of zero or one variable (Table S2).

### 3.4. Association between the Degree of Adherence to the Mediterranean Diet and Metabolic Syndrome Prevalence

An inverse relationship was found between adherence to the MedDiet and the prevalence of MetS, where good adherence to the MedDiet (≥9) was associated with a 13.9% lower prevalence of MetS (31.3%); in contrast, low adherence to the MedDiet (≤8) was associated with a higher prevalence of MetS (45.2%, *p* < 0.026; Table 1). In addition, we found that MetS was 82% more frequent among those who had low adherence to the MedDiet (score ≤ 8) compared with those who had good adherence to the MedDiet (score ≥ 9, OR: 1.817, 95%CI: 1.072–3.081, *p* = 0.027; Table 2).

### 3.5. Association between Body Mass Index Levels with the Prevalence of Metabolic Syndrome and the Degree of Adherence to the Mediterranean Diet

A linear association was observed between the BMI level and the prevalence of MetS. Namely, the prevalence of MetS in individuals with normal weight was 17.2%; in overweight individuals, it was 38.4%; and in those with obesity, it was 63.2% (*p* < 0.001; Table 2). Furthermore, the prevalence of MetS, relative to normal-weight individuals, was found to be 3.1 times more frequent in overweight individuals (OR: 3.132, 95%CI: 1.498–6.546, *p* < 0.001) and 8.3 times more frequent in obese individuals (OR: 8.287, 95%CI: 3.667–18.728, *p* < 0.001; Table 2).

In contrast, no association was found between the BMI level and the two degrees of MedDiet adherence (*p* = 0.545).

### 3.6. Association between the Components of the MEDAS-14 Questionnaire and Metabolic Syndrome Prevalence

When analysing the association between the components of the MEDAS-14 questionnaire and the prevalence of MetS, we found that in individuals who consumed two or more servings of vegetables daily, the prevalence of MetS was 24.4%, while those who consumed less than two servings of vegetables per day had a 19% higher prevalence of MetS (43.4%, *p* < 0.05; Table 1). Furthermore, MetS was 2.4 times more frequent in those who consumed less than two servings of vegetables per day compared with those who consumed two or more servings of vegetables per day (OR: 2.368, 95%CI: 1.141–4.916, *p* = 0.021; Table 2). 

In addition, individuals who consumed nuts three or more times/week had a MetS prevalence of 30.2%, while consumers who had one serving of nuts less than three times/week had a 16.6% higher prevalence of MetS (46.8%, *p* < 0.01; Table 1). 

## 4. Discussion

### 4.1. Adherence to the Mediterranean Diet

According to the results obtained in the study population, 36.4% had good adherence to the MedDiet (MEDAS-14 score ≥ 9). This result was higher than that obtained in the study of León-Muñoz et al. [20], in which a prevalence of 21.1% (MEDAS-14 score ≥ 9) was obtained in a Spanish population over 65 years of age. On the other hand, it is closer to the study conducted in an older population in the Spanish Mediterranean area, with a prevalence of 31.3% (MEDAS-14 score ≥ 8) [21]. Here, 55.3% had a medium adherence to the MedDiet (MEDAS-14 score of 8–11) and 37.9% had low adherence (MEDAS-14 score of 5–7), in agreement with the results in an elderly population in Mediterranean countries, indicating low–moderate adherence to the MedDiet in the last 10 years [22]. However, it is necessary to analyse compliance with each of the components of the MEDAS-14 questionnaire in order to understand the low prevalence of good adherence to the MedDiet.

The high percentage of individuals that consumed less than one serving per day of margarine, butter, and cream (85.2%); carbonated and/or sweetened beverages (87.1%); and red meat, hamburgers, or sausages (98.1%) can be considered positive. On the other hand, the low percentage of individuals (52.7%) who consumed less than two servings of commercial pastries (not homemade) per week was unfavourable and this decreased significantly with increasing age, which is a problem—as indicated below—in the study population, with a high prevalence of AHT (79.5%) and overweight (50.0%) or obese (25.8%), where both of these BMI ranges were associated with an increased prevalence of MetS (*p* < 0.001; Table 2). This type of ultra-processed food is rich in sugars, fats, additives, and salt [23], and has been associated with greater abdominal obesity in older people [24]. Thus, high consumption of commercial pastries may pose a risk due to the high prevalence of abdominal obesity in the study population (78.8%) and the fact that abdominal obesity, once established, is very difficult to reverse in older people [24]. It was also associated with overweight and obesity [25], AHT [26], dyslipidaemia [27], and DM2 [28], which are all variables related to the diagnosis of MetS. In addition, they were associated with a higher incidence of CVD [29]. There are several mechanisms that may facilitate the consumption of ultra-processed foods being associated with pathologies related to MetS, such as abdominal obesity [24]. This type of food, having high energy density, delays the sensation of satiety [23], leading to an increase in the portions eaten [30]. Another mechanism may be the low nutritional quality of ultra-processed foods [31].

A majority of individuals (93.6%) consumed less than one daily serving of red and/or processed meat; however, only 58.3% of the population studied preferred to consume white meat instead of red meat. This preference was higher in women (65.8%) than in men (46.6%, *p* < 0.005), which was possibly related to the fact that it is common for women to make dietary modifications with the objective of body weight control and, in this sense, it was shown that they tend to decrease red meat consumption relative to men [32]. Frequent consumption of red meat is an inadvisable dietary habit, as it has been associated with increased risks of MetS [33] and DM2 [34], which are related to the high amounts of total and saturated fats that increase the risk of obesity, hyperinsulinemia, and hyperglycaemia [35]. In addition, red meat contains heme iron, a strong pro-oxidant that promotes oxidative stress, which can damage various tissues, such as pancreatic beta cells, decreasing insulin synthesis and secretion, which ultimately affects glucose metabolism [36]. Furthermore, in processed meats, nitrate is often used as a preservative. This, in turn, is converted to nitrosamines, which affect pancreatic cell function, leading to insulin resistance [37]. In both red and processed meats, there are high levels of inflammatory mediators, such as C-reactive protein, which may be involved in increasing the risk of MetS [38]. In contrast, an inverse association was confirmed between white meat consumption and the risk of MetS and some of its components, such as hyperglycaemia and AHT [33], which could be explained in part by its lower fat content, with a higher ratio of unsaturated fatty acids and lower saturated fats [33]. 

It should be noted that 40.9% of the study population consumed less than three servings of fruit per day. This seems to be in agreement with consumption data of the Spanish population, where it was indicated that, although it is the elderly population that consumes the most fruit, Cantabria is a Spanish region where fruit consumption is lower than the national average [39]. Therefore, this population was far from the recommended consumption of this characteristic food in the MedDiet, which is rich in fibre, minerals, vitamins, and phytochemicals that, due to their antioxidant and anti-inflammatory properties, have an important role in reducing the risk of MetS [40,41]. 

The responses to the MEDAS-14 questionnaire indicated that only 20.8% of respondents consumed sofrito (tomato sauce cooked with olive oil, garlic, and onion and/or leek) two or more times a week. Lycopene, a carotenoid with strong anti-atherosclerotic, antioxidant, anti-inflammatory, antiplatelet, antihypertensive, anti-apoptotic, and protective endothelial effects, is found in tomatoes [42]. Tomato sauce with olive oil guarantees the availability of lycopene, as it is lipid-soluble [42]. In addition, its concentration is much higher than in fresh food, which is due in part to the loss of water in the cooking process [43]. It is a food that is easy to incorporate into and accepted in the diet of the elderly; therefore, it should be advised to increase its frequency of consumption, thus taking advantage of its beneficial effects in pathologies related to oxidative stress, such as MetS [44,45]. 

Olive oil consumption was habitual for cooking (98.1%); however, the majority of individuals (60.2%) did not consume an adequate amount (four or more tablespoons daily). Since the PREDIMED study [46], numerous investigations have demonstrated the importance of regular consumption of extra virgin olive oil, which is considered a functional food due to its composition being rich in monounsaturated fatty acids (MUFA), especially oleic acid and biologically active minority components, such as vitamins, minerals, polyphenols (oleuropein, hydroxytyrosol), and triterpenic acids (oleanolic and maslinic) [47,48]. Polyphenols and triterpenic acids were shown to present antioxidant [49,50] and anti-inflammatory [51] properties, suggesting the recommendation of dietary supplementation with virgin olive oil in elderly people with elevated oxidative stress status and chronic inflammation associated with age and/or pathologies, such as in MetS [52].

Only 36.0% consumed legumes three or more times a week, despite the fact that, according to data on legume consumption in Spain, the elderly are those who consume the most per person per year, with Cantabria being the region that exceeds the Spanish average consumption by 43% [39]. This can be explained by the fact that the majority of this population (72.7%) consumed 2–4 servings/week of legumes, which is in line with the criteria of Sociedad Española de Nutrición Comunitaria (SENC) [53]. The nutritional properties of legumes have been related to the prevention and control of obesity [54], DM2 [55], and MetS [56] and thus, their consumption should be increased in older people, with emphasis on the lower socio-economic class, as this is the level with the lowest consumption [39], despite the high quality and low cost of this food. In this sense, legumes are rich in fibre, which contributes to decreasing the energy density and glycaemic response; in proteins, which induce satiety; vitamins of the B group; minerals such as iron, calcium, and potassium; and bioactive components with antioxidant, anti-inflammatory, and antimicrobial properties, such as phytochemicals (enzyme inhibitors, phytohemagglutinins/lecithins, phytoestrogens, oligosaccharides, saponins, and phenolic compounds), while having a low fat content [57]. 

Fish is a characteristic food of the MedDiet, which provides a variety of nutrients. Blue fish is rich in omega-3 polyunsaturated fatty acids, such as eicosapentaenoic acid (EPA) and docohexaenoic acid (DHA), which were shown to promote anti-inflammatory properties by modulating pro-inflammatory cytokines (TNF-α and IL-6) [58]. In addition, the high protein content of white fish has been associated with a reduction in body weight through increased satiety [59] and the regulation of lipid metabolism [60]. They also provide vitamin D—the deficiency of which has been associated with an increased risk of MetS [61]—as well as iodine, selenium, and taurine. These characteristics of fish indicate that it should be included in a healthy diet, as it was shown to have beneficial effects on the variables included in MetS [62] and in the prevention of CVD [63]; however, only half of the individuals studied (50.4%) consumed the three or more servings of fish/seafood recommended for good adherence to the MedDiet.

Only 31.8% of the elderly drank seven or more glasses of wine per week, frequently related to the medical advice not to consume alcohol in this (usually polymedicated) age group, with higher consumption in men (40.8%) compared with women (26.1%, *p* < 0.05); this was possibly associated with a higher classic consumption at these ages in men. However, although optional, responsible and moderate consumption has been recommended (maximum of two glasses of wine/day in men and 1–1.5 glasses of wine/day in women) [64] and has even been associated with 24% of the health effects of the MedDiet [65]. Regular consumption should not be encouraged for teetotalers or those who should not drink wine on medical advice, although some authors have argued that moderate wine consumption has beneficial effects in the prevention of CVD [66,67], obesity [68], and diabetes [69]. 

A total of 59.8% consumed less than three servings of nuts per week, moving away from the recommendation (≥3 servings/week). As such, consumption should be increased, as it has been shown to have an inverse relationship with MetS and its components [70,71]. 

It is important to consider the beneficial effects of the diet as a whole [72]; however, more than 50% of the studied population did not consume vegetables, homemade tomato sauce, wine, legumes, oil, and nuts in the recommended amounts and frequency, which may explain why 63.6% of the population studied had low adherence (MEDAS-14 score ≤ 8) to the MedDiet.

### 4.2. Prevalence of Metabolic Syndrome

The prevalence of MetS was 40.2% according to the IDF criteria [15]. In the Spanish population over 65 years of age, according to the ENRICA study [73], the prevalence of MetS was similar (42.3%), and was quite distant from that obtained in other parts of the world, such as Mexico (72.9%) [74] and the United States (54.9 ± 1.7%) [75].

In the study population, it was found that the prevalence of MetS increased with BMI (*p* < 0.001) (Table 2). On the other hand, it has been recognised that the increasing prevalence of MetS in older people is influenced by gender, mainly associated with an altered testosterone/oestrogen balance [76]. This motivates a decrease in oestrogens in older women, leading to adipocyte hypertrophy and an increase in abdominal obesity, as associated with insulin resistance and an inflammatory and prothrombotic state [77]. In contrast, in our study, no significant differences by gender were observed in terms of abdominal obesity, although it was elevated in both women (79.5%) and men (77.7%; Table S2). However, a higher prevalence of MetS was manifested in men (47.6%) relative to women (35.4%, *p* < 0.05; Table S2), despite a majority of countries presenting a higher prevalence in women [70]. In this regard, MetS was found to be 84% more frequent in men than in women (prevalence ratio: 1.84, 95%CI: 1.39–2.45, *p* < 0.001), which was associated with a significantly higher prevalence of MetS in normal and obese men [70]. This may be related to the higher percentage of men with two, three, or four diagnostic variables of MetS relative to women (Table S2), as well as various lifestyle factors.

### 4.3. Association between Adherence to the Mediterranean Diet and the Prevalence of Metabolic Syndrome

We found that MetS was 82% more frequent among those who had low adherence to the MedDiet (score ≤ 8) compared with those who had good adherence (score ≥ 9, OR: 1.817, 95%CI: 1.072–3.081, *p* = 0.027; Table 2). Similar results were obtained in the cross-sectional study by Babio et al. [78], which was conducted with people between 55 and 80 years of age and at high cardiovascular risk, where individuals with higher scores (MEDAS-14 score ≥ 9) had a lower risk of MetS (OR: 0.44, 95%CI: 0.27–0.70, *p* < 0.001) compared with those with lower scores on the questionnaire (MEDAS-14 score < 7).

As indicated above, several foods and nutrients have been associated with MetS, but each separate food has not been proposed as solely responsible, and it is possible that the diversity of healthy foods may have an additive effect [62]. Similarly, it appears that the consumption of healthy foods with unhealthy foods could interact with each other, altering the expected response [79]. In this sense, it was shown that greater adherence to the MedDiet is associated with a lower risk of developing or progressing diet-related chronic diseases that are common in this age group, such as MetS [80]. The components of this type of healthy diet can improve insulin sensitivity, increase antioxidant capacity, and reduce metabolic disorders [81]. For all these reasons, this easy-to-follow dietary pattern, whose benefits are established soon after habitual consumption [82], should be considered one of the first treatment strategies for the prevention and treatment of MetS in the elderly [83]. In addition, adherence to the MedDiet avoids frequent monotonous diets in the elderly [84], thus facilitating the provision of nutrients adequate to their needs. 

### 4.4. Association between the Components of the MEDAS-14 Questionnaire and the Prevalence of Metabolic Syndrome

When analysing each of the components of the MEDAS-14 questionnaire, we found that MetS was 2.4 times more frequent in those who consumed less than two servings of vegetables daily compared with those who consumed two or more servings of vegetables per day (OR: 2.368, 95%CI: 1.141–4.916, *p* = 0.021; Table 2).

Vegetable consumption according to the recommendations was low (17.0%), coinciding with the consumption data of the Spanish population, where it was indicated that, although it is the elderly population that consumes the most vegetables, Cantabria is the Spanish region where vegetable consumption is the lowest [39]. This also coincides with the results of the PREDIMED-Plus study [85], according to which vegetable consumption in the northern area was significantly lower than in the other areas studied. Vegetable consumption was higher in women (21.1%) relative to men (10.7%, *p* < 0.05; Table S1), which could be related to the fact that it is common for women to make dietary modifications with the objective of body weight control, significantly raising their vegetable consumption relative to men [34]. The nutritional composition of vegetables, due to their richness in fibre, high vitamin content (mainly A, B, and C), and low fat content, have been associated with a lower risk of MetS [86,87,88]. In addition, they are rich in minerals (Se and K), as well as bioactive components with antioxidant (carotenoids and tocopherols) and anti-inflammatory (flavonoids, glucosinolates, and isothiocyanates) properties, which can modulate the inflammatory status (IL-6) in older people; as such, higher consumption should be promoted in this population group [89]. 

There were several limitations, as this study was observational, descriptive, and cross-sectional; this, in itself, posed limitations for drawing cause–effect conclusions, as correlational analysis only compares the frequencies of two variables in the same period. Therefore, longitudinal studies are needed to confirm the presented conclusions. In addition, the results of the study cannot be extrapolated to the general population of elderly people, as geographic location influences eating habits [85]; however, the results provide adequate information regarding adherence to the MedDiet in the elderly population of Santander, as most of the participants were of Cantabrian origin. This research should be repeated in order to assess whether geographical location influences the effect of dietary habits on the risk of developing MetS. Another limitation is that physical activity and other biochemical, somatometric, or nutritional variables other than those indicated in the study, which could be related to MetS, were not quantified.

## 5. Conclusions

We found that in elderly people with low adherence to the MedDiet, MetS was 82% more frequent than in those with good adherence to the MedDiet. Given that 63.6% had low adherence to the MedDiet, it is necessary to influence the elderly to increase their adherence to the MedDiet, especially in terms of the consumption of vegetables, due to its implications regarding pathologies of high prevalence in this population group, such as MetS (40.2%).

## Figures and Tables

**Table 1:** Adherence to the Mediterranean diet and the components of the MEDAS-14 questionnaire according to the prevalence of metabolic syndrome.

	NMetS *n* = 158 (59.8%)	MetS n = 106 (40.2%)	
	n (%)	n (%)	*p*-Value ^1^
**Adherence to the MedDiet**			
Good (MEDAS-14 score ≥ 9) Low (MEDAS-14 score ≤ 8)	66 (68.7) 92 (54.8)	30 (31.3) 76 (45.2)	0.026
**Adherence MedDiet (MEDAS-14 questionnaire)**			
1. Do you use olive oil as your main source of cooking fat?	156 (60.2)	103 (39.8)	0.361
2. Do you consume 4 or more tablespoons of oil per day (including oil used for frying, dressing, and meals away from home?	63 (60.0)	42 (40.0)	0.967
3. Do you consume 2 or more servings of vegetables per day? Count side and half portions as ½ point; a full portion is 200 g. Consume <2 servings of vegetables /day	34 (75.6) 124 (56.6)	11 (24.4) 95 (43.4)	0.018
4. Do you eat 3 or more pieces of fruit (including freshly squeezed juice) per day?	98 (62.8)	58 (37.2)	0.236
5. Do you eat less than 1 serving per day of red meat, hamburgers, or sausages? One portion: 100–150 g.	148 (59.9)	99 (40.1)	0.929
6. Do you consume less than 1 serving (12 g) of butter, margarine, or cream per day?	135 (60.0)	90 (40.0)	0.904
7. Do you consume less than 1 serving of carbonated and/or sweetened beverages per day?	140 (60.9)	90 (39.1)	0.379
8. Do you drink wine? Do you drink 7 or more glasses (100 mL) per week?	50 (59.5)	34 (40.5)	0.941
9. Do you consume 3 or more servings (150 g) of legumes per week?	60 (63.2)	35 (36.8)	0.411
10. Do you consume 3 or more portions of fish/seafood per week (100–150 g of fish, 4–5 pieces, or 200 g of seafood)?	83 (62.4)	50 (37.6)	0.393
11. Do you consume less than 2 servings per week of commercial (not homemade) pastries, such as biscuits and cakes?	83 (59.7)	56 (40.3)	0.962
12. Do you eat nuts 3 or more times a week (1 portion: 30 g)? Consume nuts <3 times/week	74 (69.8) 84 (53.2)	32 (30.2) 74 (46.8)	0.007
13. Do you prefer to eat chicken, turkey, or rabbit instead of beef, pork, hamburgers, or sausages?	95 (61.7)	59 (38.3)	0.471
14. Do you eat cooked vegetables, pasta, rice, or other foods with sofrito (tomato sauce simmered with olive oil, garlic, and onion and/or leek) 2 or more times a week?	30 (54.5)	25 (45.5)	0.367

NMetS: no metabolic syndrome; MetS: with metabolic syndrome; ^1^ Pearson chi-square.

**Table 2:** Association between the degree of adherence to the Mediterranean diet, vegetable consumption, and body mass index level with the prevalence of metabolic syndrome.

	NMetS *n* = 158 (59.8%)	MetS *n* = 106 (40.2%)			
	*n* (%)	*n* (%)	*p*-Value ^1^	OR (95%CI)	*p*-Value ^2^
Good adherence to the MedDiet (≥9 MEDAS-14)	66 (68.7)	30 (31.3)	0.026		0.027
Low adherence to the MedDiet (≤8 MEDAS-14)	92 (54.8)	76 (45.2)		1.817 (1.072–3.081)	
≥2 servings vegetables/day	34 (75.6)	11 (24.4)	0.018		0.021
<2 servings vegetables/day	124 (56.6)	95 (43.4)		2.369 (1.141–4.916)	
BMI categories:			<0.001		<0.001
Normal weight	53 (82.8)	11 (17.2)			
Overweight	80 (60.6)	52 (39.4)		3.132 (1.498–6.546)	
Obese	25 (36.8)	43 (63.2)		8.287 (3.667–18.728)	

NMets: no metabolic syndrome; MetS: with metabolic syndrome; BMI: body mass index. ^1^ Pearson chi-square; ^2^ Wald chi-square. OR: odds ratio; 95%CI: 95% confidence interval.

## Data Availability

The data presented in this study are available on request from the corresponding author.

## Supplementary Materials

The following supporting information can be downloaded from https://www.mdpi.com/article/10.3390/nu14214536/s1. Table S1: Adherence to the Mediterranean diet and the components of the MEDAS-14 questionnaire by gender; Table S2: Sociodemographic and health-related variables by gender.
