# Supplementary material for: Adherence to the Mediterranean Diet Is Inversely Associated with the Prevalence of Metabolic Syndrome in Older People from the North of Spain
Source: Nutrients. 2022 Oct 28;14(21):4536. doi: 10.3390/nu14214536 (PMC9657522; doi:10.3390/nu14214536)
Supplement: Supplementary file 1 [file nutrients-14-04536-s001.zip › nutrients-1931491-supplementary.pdf]

Supplementary Materials

Table S1. Adherence to the Mediterranean Diet and the components of the MEDAS-14 questionnaire by gender

	Men (N=103)	Women (N=161)		Total (N=264)
	n (%)	n (%)	p[1]	n (%)
**Adherence to MedDiet (4 levels)**				
High (12-14)	2 (1.9)	3 (1.9)		5 (1.9)
Medium (8-11)	54 (52.4)	92 (57.1)	0.874	146 (55.3)
Low (5-7)	41 (39.8)	59 (36.6)		100 (37.9)
Very low (<5)	6 (5.8)	7 (4.3)		13 (4.9)
**Adherence to MedDiet (2 levels)**				
Good (≥ 9)	35 (34.0)	61 (37.9)	0.520	96 (36.4)
Low (≤ 8)	68 (66.0)	100 (62.1)		168 (63.6)
**Adherence to MedDiet (MEDAS-14 Questionnaire)**				
1-Use olive oil as the main source of cooking fat?	101 (98.1)	158 (98.1)	0.964	259 (98.1)
2- Do you consume 4 or more tablespoons of oil daily (including oil used frying, dressing or meals away from home, etc.?	42 (40.8)	63 (39.1)	0.790	105 (39.8)
3-Do you consume 2 or more servings of vegetables per day? Count side and half portions as ½ point; a full portion is 200g.	11 (10,7)	34 (21.1)	*0.028*	45 (17.0)
4-Do you eat 3 or more pieces of fruit (including freshly squeezed juice) per day?	57 (55.3)	99 (61.5)	0.321	156 (59.1)
5-Do you eat less than 1 serving per day of red meat, hamburgers or sausages? One portion: 100-150g.	94 (91.3)	153 (95.0)	0.224	247 (93.6)
6-Do you consume less than 1 serving (12g.) of butter, margarine or cream per day?	88 (85.4)	137 (85.1)	0.939	225 (85.2)
7-Do you consume less than 1 serving of carbonated and/or sweetened beverages per day?	89 (86.4)	141 (87.6)	0.782	230 (87.1)
8- Do you drink wine? Do you drink 7 or more glasses (100ml) per week?	42 (40.8)	42 (26.1)	*0.012*	84 (31.8)
9-Do you consume 3 or more servings (150g.) of legumes per week?	42 (40.8)	53 (32.9)	0.194	95 (36.0)
10- Do you consume 3 or more portions of fish/seafood per week (100-150g. of fish, 4-5 pieces or 200. of seafood)?	52 (50.5)	81 (50.3)	0.978	133 (50.4)
11- Do you consume less than 2 servings per week of industrial (not homemade) confectionery such as biscuits and cakes?	50 (48.5)	89 (55.3)	0.285	139 (52.7)
12- Do you eat 3 or more times a week nuts (1 portion: 30g.)	38 (36.9)	68 (42.2)	0.388	106 (40.2)
13- Do you prefer to eat chicken, turkey, rabbit instead of beef, pork, hamburgers or sausages?	48 (46.6)	106 (65.8)	*0.002*	154 (58.3)
14- Do you eat 2 or more times a week cooked vegetables, pasta, rice or other foods with sofrito (tomato sauce, simmered with olive oil, garlic, onion and /or leek?	25 (24.3)	30 (18.6)	0.271	55 (20,8)

[1]Pearson´s chi-square

Table S2. Sociodemographic and health-related variables by gender

	Men (N = 103)	Women (N = 161)	p-value	Total (N = 264)
	N (%)	N (%)		N (%)
**Age groups**			0.833*	
**65-69**	36 (34.9)	52 (32.3)		88 (33.3)
**70-74**	36 (34.9)	62 (38.5)		98 (37.1)
**75-79**	31 (30.2)	47 (29.2)		78 (29.6)
**Marital status**				
**Married/partnered**	79 (76.7)	88 (54.7)	<0.001*	167 (63.2)
**Separated**	7 (6.8)	9 (5.6)		16 (6.1)
**Widowed**	7 (6.8)	41 (25.4)		48 (18.2)
**Single**	10 (9.7)	23 (14.3)		33 (12.5)
**Type of cohabitation**				
**Couple**	80 (77.7)	84 (52.2)	=0.001*	164 (62.1)
**With relatives**	5 (4.8)	24 (14.9)		29 (11.0)
**With a carer**	1 (1.0)	2 (1.2)		3 (1.1)
**Alone**	17 (16.5)	50 (31.1)		67 (25.4)
**Shared flat**	0 (0.0)	1 (0.6)		1 (0.4)
**Educational level**				
**University**	54 (52.4)	53 (32.9)	= 0.002*	107 (40.5)
**Secondary school**	28 (27.2)	46 (28.6)		74 (28.0)
**Primary school**	21 (20.4)	55 (34.2)		76 (28.8)
**Incomplete**	0 (0.0)	7 (4.3)		7 (2.7)
**Who cooks**				
**Partaker**	23 (22.4)	140 (86.9)	<0.001*	163 (61.7)
**The couple**	56 (54.4)	3 (1.9)		59 (22.3)
**Relatives**	20 (19,4)	15 (9.3)		35 (13.3)
**Carer**	2 (1.9)	3 (1.9)		5 (1.9)
**Catering**	2 (1.9)	0 (0.0)		2 (0.8)
**BMI categories**			0.001*	
**Normal weight**	13 (12.6)	51 (31.7)	<0.001**	64 (24.2)
**Overweight**	62 (60.2)	70 (43.5)	0.008**	132 (50.0)
**Obesity**	28 (27.2)	40 (24.8)	0.664**	68 (25.8)
**Abdominal obesity**	80 (77.7)	128 (79.5)	0.722*	208 (78.8)
**Health status**				
**Hypertension**	89 (86.4)	121 (75.2)	0.027**	210 (79.5)
**Hyperglycaemia**	47 (45.6)	37 (23.0)	<0.001**	84 (31.8)
**Hipertriglyceridemia**	21 (20.4)	38 (23.6)	0.541**	59 (22.3)
**Low-HDL**	21 (20.4)	31 (19.3)	0.821**	52 (19.7)
**Number of variables MetS**			<0.001*	
**none**	6 (5.8)	32 (19.9)	0.002**	38 (14.4)
**1**	28 (27.2)	74 (46.0)	0.002**	102 (38.6)
**2**	37 (35.9)	36 (22.4)	0.017**	73 (27.6)
**3**	23 (22.3)	17 (10.6)	<0.001**	40 (15.2)
**4**	9 (8.7)	2 (1.2)	0.01**	11 (4.2)
**Metabolic syndrome**	49 (47.6)	57 (35.4)	0.049*	106 (40.2)

*Pearson Chi-square test **Pearson Chi square Goodness-of-Fit test
